# Association of Dietary Patterns with Cardiovascular Disease Risk Factors in Mexican Adults: Insights from a Cross-Sectional Descriptive Study

**DOI:** 10.3390/nu16060804

**Published:** 2024-03-12

**Authors:** Alejandra Vázquez-Aguilar, Ascensión Rueda-Robles, Lorenzo Rivas-García, Héctor Vázquez-Lorente, Carmen María Duque-Soto, Karla Lizbet Jiménez-López, Isabel Cristina Marín-Arriola, Martha Alicia Sánchez-Jiménez, Patricia Josefina López-Uriarte

**Affiliations:** 1 Institute of Nutrition and Food Technology “Jose Mataix Verdú”, Biomedical Research Center, University of Granada, 18016 Armilla, Spain; alejandravqz@correo.ugr.es (A.V.-A.); lorenrivas@ugr.es (L.R.-G.); hectorvazquez@ugr.es (H.V.-L.); 2 Department of Food Science and Nutrition, University of Granada, Campus Universitario s/n, 18071 Granada, Spain; ruedarobles@ugr.es (A.R.-R.); carmenduque@ugr.es (C.M.D.-S.); 3 Department of Physiology, Schools of Pharmacy and Medicine, University of Granada, 18071 Granada, Spain; 4 Department of Social Sciences, Southern University Center, University of Guadalajara, Ciudad Guzmán 49000, Mexico; lizbet.jimenez@cusur.udg.mx; 5 Department of Promotion, Preservation and Development of Health, Southern University Center, University of Guadalajara, Ciudad Guzmán 49000, Mexico; cristinam@cusur.udg.mx (I.C.M.-A.); martha.sjimenez@alumnos.udg.mx (M.A.S.-J.); 6 UDG-CUSUR-CA-1051, Quality of Life, Gender, and Food Processes, Southern University Center, University of Guadalajara, Ciudad Guzmán 49000, Mexico; 7 Department of Exact Sciences and Methodologies, Southern University Center, University of Guadalajara, Ciudad Guzmán 49000, Mexico

**Keywords:** dietary patterns, Mexican adults, overweight, obesity, cardiovascular disease risk factor

## Abstract

Dietary patterns (DPs) are an essential tool to analyze the relationship between diet and health as they have presented an association with the incidence of chronic non-communicable diseases. Therefore, the aim of this study was the identification and characterization of DPs and their association with cardiovascular risk factors. For this purpose, a cross-sectional descriptive study was carried out in 165 Mexican adults, including dietary intakes derived from a validated food frequency questionnaire, clinical history, anthropometry, and biochemical biomarkers using standardized procedures for glucose, total cholesterol, triglycerides, LDL-c, and HDL-c. DPs were identified through principal component analysis and ordinal logistic regression was used to examine associations between DPs and cardiovascular disease risk factors. Three DPs were identified: Mexican Fast-Food, Variety-Food, and Healthy-Economic, with a high prevalence of overweight and obesity (78%). Having a high adherence to a Mexican Fast-Food pattern (OR 1.71 CI 1.4–2.8), being sedentary (OR 4.85 2.32–10.15) and smoking (0R 6.4 CI 2.40–16.9) increased the risk of having a high scale of risk factors (four or more risk factors simultaneously). In conclusion, the Mexican Fast-Food pattern showed an increase in the risk of having multiple risk factors, while a sedentary lifestyle and overeating were largely responsible for the prevalence of overweight and obesity in this group of Mexican adults.

## 1. Introduction

In recent years, dietary patterns (DPs) have become an essential tool for the analysis of diet and its association with the risk of developing chronic diseases [1,2]. Unhealthy diets have been linked to the high prevalence and development of overweight and obesity, cardiovascular disease (CVD), and insulin resistance, which leads to type 2 diabetes mellitus (DM2) [3]. In this sense, it is estimated that 90% of CVD and DM2 cases in the Mexican population are a result of overweight and obesity [4].

According to the National Health and Nutrition Survey in Mexico, the prevalence of obesity increased by 12.35% from 2012 to 2020. In fact, Mexico is the country with the second-highest prevalence of obesity in the adult population worldwide. More than 70% of Mexican adults over 20 years of age are overweight, this incidence being higher in men than in women, while the prevalence of obesity is usually higher in women than in men [5].

There is strong scientific evidence demonstrating that consuming healthy DPs is associated with a lower risk of CVD [2]. These DPs are typically defined by the regular consumption of vegetables, fruits, whole grains, low-fat dairy, fish, and shellfish. Conversely, they involve reducing intake of red and processed meats, refined grains, and sweetened foods and beverages. Additionally, the inclusion of nuts and moderate alcohol consumption have also been identified as beneficial components of a healthy DP [6]. Likewise, results from clinical studies of dietary interventions have suggested a significant impact of these DPs on cardiovascular disease risk, dyslipidemias, and blood pressure [6].

Results of different studies carried out in the Mexican population, both in children and adults, revealed that the main foods consumed belong to diverse DPs, such as Westernized (defined by the consumption of ultra-processed foods, commercial food, red meat, and sugary drinks), healthy (characterized by the consumption of fruits, vegetables, white meats such as chicken and fish, grains, and whole grains), and rural or traditional (including consumption of tortillas and foods derived from corn, legumes, vegetables, and small amounts of foods of animal origin such as chicken and eggs) [7,8,9,10]. Thus, results have shown an association and adherence to the consumption of Westernized DPs with a greater probability of developing CVD and metabolic disorders [8]. On the contrary, adherence to healthy DPs has been associated with a lower risk of presenting high lipid concentrations in the blood [10]. The incidence of dyslipidemia, a disease characterized by high concentrations of total cholesterol (TC) and low-density lipoproteins (LDL-c) along with low concentrations of high-density lipoproteins (HDL-c), leads to the accumulation and oxidation of LDL-c in the arterial wall. This phenomenon has been associated with an increased risk of atherosclerotic disease, including coronary artery disease and stroke [11]. Additionally, higher abdominal obesity is associated with hyperglycemia, insulin resistance, hyperlipidemia, and low HDL-c concentrations [12], while the LDL-HDL-c ratio strongly predicts atherosclerosis progression [13].

In the current challenging landscape for public health, emphasizing the promotion of a healthy lifestyle and the formulation of policies and strategies to address the aforementioned epidemiological situation in Mexico, this study aimed to delineate and analyze various DPs and their correlation with cardiovascular disease risk factors among a cohort of Mexican adults.

## 2. Materials and Methods

### 2.1. Study Design and Volunteers

A flow chart of the participants enrolled in the present study is represented in the Supplementary Material (Figure S1). Briefly, approximately 4500 adults from a company located in Zapopan, Jalisco, Mexico, were invited to participate in the present study through an information campaign. In the recruitment phase, 444 volunteers were registered; 231 were contacted by telephone and agreed to make an appointment at the company’s medical office to have a personal interview to explain the study, clarify doubts, sign the informed consent form, and verify compliance with the criteria. The procedures and methods used to measure and collect anthropometric, biochemical, clinical, and dietary data were explained to each volunteer orally and in writing, and those who agreed to participate signed the informed consent and provided general data. After a thorough evaluation, it was determined that 165 adults should meet the required criteria: (I) body mass index (BMI) ≤ 35; (II) not suffering from any chronic disease or maintaining anti-inflammatory drug treatment, hormones, or corticosteroids; (III) no drug or alcohol abuse; and (IV) not being pregnant or lactating. The methodology used in this study was approved by the Ethics and Research Committee of the University Center for Health Sciences at the University of Guadalajara (reference CI-02613). The 165 participants included in this study were summoned to an interview in which the sociodemographic, clinical, and dietary questionnaires were completed and the anthropometric measurements were obtained (Supplementary Material Figure S1)

### 2.2. Variables and Data Collection

#### 2.2.1. Clinical History

A questionnaire was administered to the participants to collect their sociodemographic data, including age, marital status, education, lifestyle habits, and history of chronic diseases.

#### 2.2.2. Anthropometric Measurements

Body weight was measured using a TANITA^®^ digital scale (model UM-061, TANITA, Amsterdam, The Netherlands) with 0.1 kg accuracy. Participants were instructed to wear light clothing and asked to remove their shoes. Height was measured using a SECA^®^ portable wall stadiometer (SECA, Hamburg, Germany), placing the individual in a standing position and verifying that the head complied with the Frankfort plane, and the BMI value [weight (kg)/height^2^ (m)] was subsequently obtained. The BMI cut-off points were defined in accordance with the criteria set by the World Health Organization (WHO) as follows: <18.5, indicating underweight; 18.5–24.9, classified as normal; 25–29.9, categorized as overweight; and ≥30.0–34.9, indicating obesity I [14]. To measure waist circumference, a Gülick^®^ (Leverkusen, Germany) flexible vinyl tape was used, with an accuracy of 0.1 cm; participants were asked to stand, locating the waist at the midpoint between the upper edge of the iliac crest and below the last floating rib; after a normal expiration, the data were obtained in triplicate and expressed in centimeters. Abdominal obesity was subsequently defined according to the criteria of the International Diabetes Federation (IDF) (i.e., ≥80 cm for women and ≥90 cm for men) [15].

#### 2.2.3. Physical Activity

A validated questionnaire was applied to determine the level of physical activity [16]. Participants were asked about the time (min) and frequency (days/week) that they performed physical activity, such as walking, dancing, or climbing stairs, or whether they performed physical conditioning exercises or a particular sport. The total time/week was obtained, and the level of physical activity was determined based on the recommendations of the WHO (2022) [17] to avoid a sedentary lifestyle, which states that moderate physical activity should be carried out at least 150 to 300 min/week.

#### 2.2.4. Diet and Pattern Analysis

A 165-item semiquantitative food frequency questionnaire (FFQ) was applied, which was validated in 97 Mexican adults (men and women) [18]. The authors of this FFQ evaluated its reproducibility and validity for nutrients and food groups, obtaining results suitable to determine the average daily energy and nutrient intake in the Mexican adult population [18]. Each participant was asked the amount and frequency (number of times in each period) that they usually consumed each food; responses ranged from “never or rarely” to “six or more times a day”. The responses obtained from each item were converted to energy/day (kcal/d) and grams per day (g/d). In addition, foods and beverages were classified into food groups according to their nutritional content, taking Mexican food composition tables as references [19] and obtaining 22 food groups (Table S3, Supplementary Materials). These groups were used to perform the principal component analysis (PCA) and to identify the DPs most consumed by the study participants. Foods that obtained very low consumption responses in the FFQ (less than 10%) were eliminated, which corresponded to semi-skimmed milk, cottage cheese, lamb meat, rabbit meat, asparagus, olives, a mixture of different oils, safflower oil, rosé wine, aged red wine, young red wine, white wine, and liqueurs.

### 2.3. Blood Extraction and Determination of Biomarkers

Personnel from an accredited laboratory performed the blood collection. After 12 h of fasting, blood samples were extracted by venipuncture in vacuum tubes using a vacutainer system and stored in containers at 4 °C. They were then transferred to the laboratory for processing the same day. Plasma was separated by centrifugation at 3500 rpm for 10 min and transferred to properly labeled Eppendorf (Hamburg, Germany) tubes for storage. Subsequently, each analyte was measured according to the method established by the manufacturer for each of the different reagents, namely glucose, total cholesterol, triglycerides, LDL-c, and HDL-c, which were provided by the SPINREACT brand, Barcelona, Spain. Quality control was performed jointly by observing the characteristics of the reagent and pathological control for each analyte to ensure the accuracy and precision of the results.

### 2.4. Statistic Analysis

The normality of all variables was accepted after the employment of the Kolmogorov–Smirnov test. Sociodemographic, clinical, and anthropometric variables were described according to the gender of the participants; the Chi-squared test was used for qualitative variables and Student’s *t*-test for quantitative variables. Biochemical data were described in relation to the participants’ BMI, and the ANOVA test and Bonferroni post hoc test were used. Multivariate PCA analysis was used to identify PDs, and Varimax rotation was used to maintain non-correlation and improve precision and interpretability. The factorability of the data was confirmed using the Kaiser–Meyer–Olkin (KMO) test of sampling adequacy and Bartlett’s test of sphericity, achieving a correct estimate for the statistical values of this sample. The factor loadings refer to the coefficients that define the components in each DP, and an absolute value ≥ 0.30 was established; those foods that had this value were significant enough to characterize each of the three DPs. At least five food groups defined each pattern as well as the absolute value (≥0.30) of the factor loading for the PCA components. Subsequently, tertiles were created with the factor loadings resulting from the PCA, which indicated adherence to each of the DPs identified in this analysis. Finally, ordinal logistic regression models and a generalized linear regression model were created, and Odds Ratios were calculated (95% CI) with the objective of determining adherence to DPs and its relationship with cardiovascular risk factors. The level of statistical significance for all tests was set at *p* ≤ 0.05. Data were analyzed using the SPSS Statistics v 22.0 (SPSS Inc., Chicago, IL, USA).

## 3. Results

The participants had an average age of 39 years; 74% were female; 60% of the participants were married, and their school grades were secondary level (36.1%) and high school (32.7%). When analyzing the anthropometric data, 78% of the participants were overweight or obese (Table 1).

Table 2 shows the anthropometric, biochemical, and clinical variables categorized according to BMI classification into normal weight, overweight, and obese; the data comparison was performed with normal weight versus overweight and obese, respectively. Circulating glucose concentrations were within normal ranges in the three groups, but when conducting intergroup comparisons, differences were observed between overweight and obese subjects (*p* = 0.005). Concerning triglycerides, increased levels were found in the obesity group compared to the overweight (*p* = 0.003) and normal-weight (*p* = 0.001) groups, respectively. HDL-c parameters were significantly higher in normal-weight individuals when compared to those who were overweight (*p* = 0.01) and obese (*p* = 0.006). Regarding the LDL/HDL-c ratio, significant differences were observed when comparing the normal-weight group with overweight (*p* = 0.003) and obesity (*p* = 0.02), this contrast being greater in the latter scenario. Finally, no significant differences were observed in total cholesterol, LDL-c, and systolic blood.

**Figure 1:**
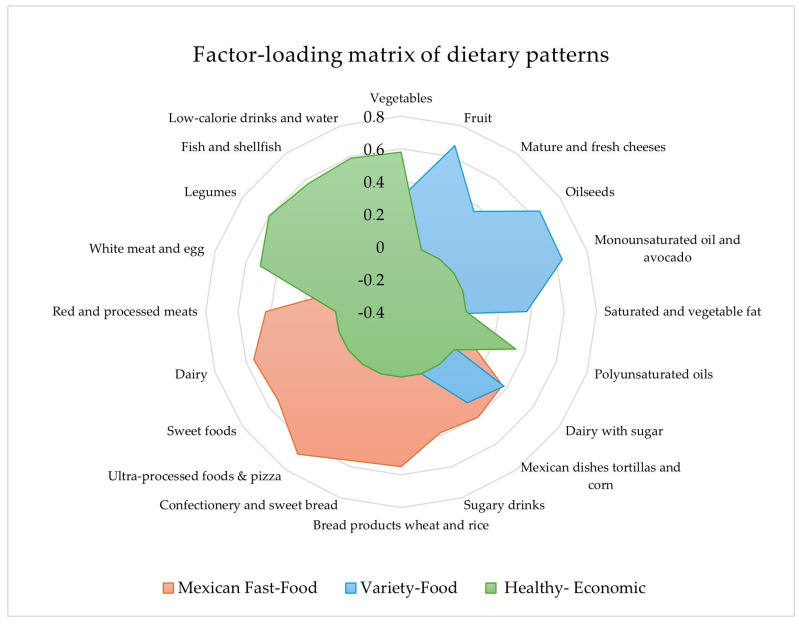
Factor-loading matrix of dietary patterns.

Figure 1 displays the food groups included in each of the three DPs identified in our study, considering a score ≥0.3 as significant in the factor-loading matrix of the food groups that make up each of the DPs, as presented in Table S1 (Supplementary Materials). The observed patterns explained 31.62% of the total variance in food consumption among the study participants. The first category, “Mexican Fast-Food”, was defined by the consumption of commercial and ultra-processed foods, including pizza, commercial pastries, sweet bread, bread and wheat-based products, rice, dairy products, dairy products with sugar, sweet foods, and beverages, red and processed meats, Mexican dishes, tortillas, and corn derivatives. The second category, which was referred to as “Variety-Food”, included the consumption of vegetables, fruits, monounsaturated and polyunsaturated oils, avocado, oilseeds (almonds, nuts, and peanuts), mature and fresh cheeses, saturated and vegetable fats, dairy with sugar, sugary drinks, Mexican dishes, tortillas, and corn derivatives. Finally, the third category, called “Healthy-Economic”, involved the consumption of vegetables, polyunsaturated oils, white meat, eggs, legumes, fish, shellfish, low-calorie drinks, and water.

The adherence to the three identified DPs and their correlation with the sociodemographic, anthropometric, and biochemical variables are depicted in Table S2 (Supplementary Materials). Among adults, a predominance of adhesion to the “Mexican Fast-Food” DP (35.2%) was observed, followed by the “Variety-Food” DP (32.7%), while the “Healthy-Economic” DP had the least adherence (32.1%). Nevertheless, no significant differences were observed between the three DPs for gender, smoking, BMI, prevalence of overweight or obesity, waist circumference, presence of abdominal obesity, biochemical parameters, and the LDL/HDL-c ratio.

The results regarding energy, macronutrients, dietetic fiber, and cholesterol intake are presented in Table 3. In this sense, the participants who adhered to the consumption of the Mexican Fast-Food DP presented the highest intake of energy (*p* < 0.01) and saturated fatty acids (*p* < 0.001) compared to those consuming the other identified DPs. On the other hand, regarding the consumption of carbohydrates, the Variety-Food DP pattern presented the highest carbohydrate (*p* = 0.05) intake. As for the consumption of polyunsaturated fatty acids (*p* < 0.001) and dietary cholesterol (*p* < 0.01), these parameters were significantly higher in the Healthy-Economic pattern compared to the other two DPs. Concerning dietary fiber, in the Variety-Food DP, a significantly greater (*p* ≤ 0.05) fiber contribution was observed (27.94 g) than in the Mexican Fast-Food (23.24 g) and Healthy-Economic (22.56 g) DPs.

**Figure 2:**
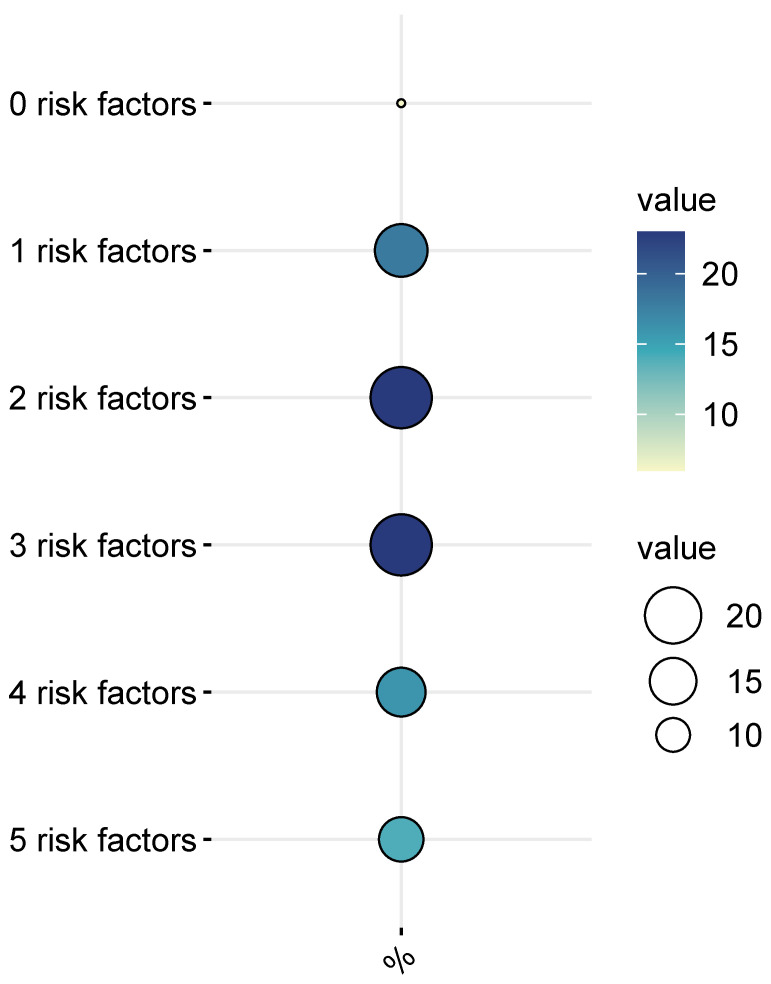
Participants of the study classified according to the summary of risk factors (%).

Table 4 displays the findings of the ordinal logistic regression model, which was used to assess the impact of compliance with three different DPs on the prevalence of cardiovascular disease risk factors (as shown in Figure 2). This study considered the following categories of cardiovascular disease risk factors: “low” (indicating a risk score of 0–1 condition), “moderate” (indicating a risk score of 2–3 conditions), and “high” (indicating a risk score of ≥4 conditions). The analysis was based on the following covariates: BMI (normal weight, overweight, obesity), abdominal obesity (≥80 for women; ≥90 for men), LDL/HDL-c ratio (≥2 with risk/≥1.9 without risk), triglycerides (≤149 mg/dL = desirable, ≥150 mg/dL = elevated), and smoking (Smoker, Non-smoker); the factors of physical activity and smoking habits were included. The model was statistically significant (X^2^ = 39.510, *p* = <0.001) and explained 25.4% (R^2^ = 0.254) of the categories of the dependent variable. Table 4 shows the results of a generalized linear model that calculated the Odds Ratios (ORs) and prediction values. The analysis found that being sedentary increases the chances of scoring high on cardiovascular disease risk factors by 4.85 times, smoking increases the chances by 6.21 times, and having a high adherence to a Mexican Fast-Food DP increases the chances by 1.71 times. However, the Variety-Food DP and the Healthy-Economic DP did not affect cardiovascular disease risk factors.

## 4. Discussion

In the present study, the dietary behaviors of a group of Mexican adults were evaluated in order to identify intake patterns and their association with an increased risk of the incidence of CVD. An exhaustive analysis of the obtained data resulted in the identification of three distinct dietary patterns labeled as Mexican Fast-Food, Variety-Food, and Healthy-Economic DPs, accounting for 31.6% of the total food consumption variance, as reported by the PCA. In this sense, the Mexican Fast-Food DP proved to be the most frequent with 35.2% adherence. Its main characteristics revolved around a higher calorie (3166 kcal/d) and saturated fat intake (31.26 g/day *p* = 0.001) than observed for the other patterns, being in line with previous literature on the dietary behavior of the Mexican population [7,10,20,21,22,23].

Thus, this prominent pattern (Mexican Fast-Food) describes an eating behavior high in commercial and ultra-processed foods, red and processed meats, and added-sugar foods, which appear to be related to poor health quality. Indeed, adherence to this type of dietary pattern has been associated with a higher risk of developing metabolic syndrome (MetS) [24]. Furthermore, research has linked the consumption of foods rich in sugars and fats with postprandial inflammation, contributing to an increase in advanced glycation end products (AGEs), which may raise concerns about the adequacy of their ingestion. However, the presence of certain antioxidants provided by a healthy and nutrient-rich diet could partially reduce the presence of these AGEs, thus decreasing the impact of these foods on postprandial inflammation and the effect on overall health [25].

This Westernization of the diet, which has been supported by recent ENSANUT 2020 data, could pose not only a health concern but also an environmental challenge for the Mexican population. Urban citizens consume a wider variety of foods related to a higher strain on natural resources, including meat [26]. The presented PCA results revealed a significant correlation of the Mexican Fast-Food DP with increased consumption of red and processed meats (0.48) and, especially, of commercial and ultra-processed foods (0.68), which is a pressing health concern. Ultra-processed foods are responsible for 30% of energy intake in Mexico, resulting in a reduction in dietary diversity and micronutrient intake as well as an increased risk for chronic disease [27,28]. This is consistent with observations in our study, where adherence to this DP resulted in a 1.7-fold increase in cardiovascular risk when compared to the other identified patterns.

On the other hand, the Variety-Food DP, with a caloric contribution of 2762.76 kcal/day (Table 3), showed a higher carbohydrate consumption compared to other dietary patterns (*p* < 0.05). Interestingly, it also showed the highest presence of dietary fiber among the studied patterns (27.94 g/day, Table 3). As opposed to previous research, our results did not associate the inherent food diversity of this pattern with an increase in overweight and obesity among the considered participants [23].

Finally, the Healthy-Economic DP had a reduced caloric contribution (2624 kcal/day) while exhibiting the highest intake of polyunsaturated fatty acids (22.65 g/day, *p* = 0.001) and total dietary cholesterol (383.52 mg/day, *p* = 0.01) (Table 3). The healthy DP, also known as prudent, has been commonly described in various populations [29,30,31] and has been associated with the prevention of CVD and MetS [7,24]. However, in the present results, the adherence to this DP did not show an association with a lower prevalence of overweight or obesity. Conversely, higher BMI and abdominal obesity were observed in participants who adhered to this pattern (Table S2) compared with other DPs, but it did not reach statistical significance. This may be related to behavioral changes leading to an improvement in the quality of their diet as a result of being confronted with high BMI results. Indeed, these results agreed with previous literature on healthy adults, in which those who adhered to a healthy pattern presented a higher percentage of body fat [10].

In our study, obese participants had high levels of triglycerides and lower levels than recommended of HDL-c (Table 2), factors known to increase cardiovascular risk and lead to insulin resistance development [32,33]. Moreover, our study revealed that 73% of participants were leading sedentary lifestyles (Table 1), highlighting yet another modifiable risk factor for cardiovascular diseases. A sedentary lifestyle not only escalates the risk of all-cause mortality but also doubles the likelihood of developing cardiovascular disease, diabetes, and obesity [34].

According to the WHO, healthy calorie intake in adults should be around 2000 kcal/day [35]. However, the observed caloric contribution of the three DPs studied largely exceeds this value, being up to 1166 kcal above the recommendation (Table 3). The observed difference between consumed kcal and energy expended appears to be one of the main causes of the increase in overweight and obesity. This phenomenon is usually related to the combined effect of both overconsumption of high-calorie foods found in modern diets and a sedentary lifestyle [36], which were confirmed for the Mexican population by the evidence recorded in the present study. Particularly, research has shown the importance of the reduction in physical activity among Westernized countries as a determinant factor for the prevalence of these conditions [37,38] where the lifestyle is increasingly defined by a more sedentary approach to work and leisure time, requiring increasingly less physical effort [39,40]. Indeed, this is further observed in our study, where most of the studied subjects were office workers, spending a significant amount of their 8 h shift sitting.

Regarding the LDL/HDL-c ratio, overweight and obese individuals had significantly higher levels of this parameter (2.60, *p* = 0.001) as compared to the normal-weight group, with a value above the recommended specification of ≤2 (Table 2). On this basis, a cut-off value of 2.5 for the LDL/HDL-c ratio has been proposed as a predictive marker for cardiovascular disease risk [41]. Additionally, according to the United States Department of Agriculture, cholesterol intake should not exceed 300 mg/day in the habitual diet in order to avoid developing dyslipidemia [42]. In our study, both the Mexican Fast-Food and the Healthy-Economic DPs had the highest contribution in terms of total cholesterol in the diet, with 366 mg/dL/day and 383 mg/dL/day, respectively (Table 3).

Overweight and obese participants in our study did not meet sufficient criteria for a MetS diagnosis [15]. However, previous evidence has suggested an increased tendency in obese individuals towards low-grade inflammation mediated by pro-inflammatory markers from the adipose tissue, which have been related to insulin resistance and metabolic disorders and were not observed in normal-weight subjects [25]. Even though these biomarkers were not evaluated in the present study, it should be highlighted that a significant proportion of the overweight and obese participants presented multiple concurrent cardiovascular risk factors, with over 49.6% exhibiting three or more of them (Figure 2). This observation implies an increased likelihood of subclinical inflammation among individuals with excess adipose tissue. Furthermore, the persistence of deleterious DPs and sedentary behavior in these individuals may heighten the susceptibility to metabolic disorders and insulin resistance.

Finally, an ordinal logistic regression model was created to evaluate the associations between a scale of risk factors and adherence to the three different patterns identified in this study. The resulting prediction values exhibit three fundamental aspects related to an increased scoring of cardiovascular disease risk factors: (1) smoking, with a 6.21-fold increased likelihood of scoring the highest on the cardiovascular disease risk scale (95% CI, 2.40–16.9); (2) a sedentary lifestyle, which presented a 4.85-fold increase (95% CI, 2.32–10.15); and 3) and adherence to Mexican Fast-Food DP, increasing this probability 1.71 times (95% CI, 1.04–2.80). Thus, these results confirm the relation of the adherence to a Westernized DP with an increase in the presence of cardiovascular disease risk factors. This is further supported by previous research, where the presence of dietary patterns rich in refined foods increases the risk of developing CVD [7] and the prevalence of overweight, obesity, and abdominal obesity [23]. As previously mentioned, a sedentary lifestyle is one of the main modifiable risk factors in the development of CVD and all-cause mortality [43]. In addition, consumption of tobacco has been associated by considerable epidemiological evidence with cardiovascular diseases [44]. However, the complexity of the chemical composition of cigarette smoke, which includes a mixture of more than 4000 chemical components that are distributed in particulate and gaseous phases, makes it difficult to discern the molecular mechanisms by which their effects are mediated [45].

Some limitations can be observed in this study, such as the obtention of self-reported diet information, an underestimation of food intake [46], the small sample size of male participants, and homogeneity in participants’ jobs. Nevertheless, the present study also has some important strengths that should be highlighted. In this sense, the interviews were performed by nutrition professionals who received training in the application and methodology of the FFQ, in addition to using a validated FFQ for the Mexican population, ensuring a rigorous methodology in the application of the validated questionnaire to identify eating patterns in the Mexican adult population [18]. Furthermore, a specialized software Nutricloud^®^ v 2016 [47] was used for the interpretation of this diet information, which allowed a more precise description of the diet analysis. Overall, these procedures allowed for meticulous and accurate interpretation of the obtained data for the identification of the predominant dietary patterns of Mexican adults and their impact on health.

## 5. Conclusions

In this study, three distinct dietary patterns were identified among Mexican adults, describing different approaches to eating and a different impact on cardiovascular disease risk. The Mexican Fast-Food pattern showed an increase in the likelihood of the concomitance of three or more cardiovascular risk factors. The prevalence in this group of a higher BMI was significant, related to a more sedentary lifestyle and increased overeating. On the other hand, the Variety-Food pattern showed an elevated carbohydrate intake while the Healthy-Economic pattern was characterized by a higher consumption of polyunsaturated fatty acids and total dietary cholesterol.

These findings elucidate the diversity of the dietary profiles in Mexican adults and their potential implications on health in this population, paving the way for future research furthering the implications of behavioral aspects and the importance of epigenetic studies focused on the development and perpetuation of overweight and obesity conditions.

## Figures and Tables

**Table 1:** Sociodemographic characteristics of the participants included in this study.

Variables	Men (n = 42)	Women (n = 124)	Total (n = 165)	*p*
Age (years), M ± SD	36.24 ± 9.15	40.13 ± 9.69	39.9 ± 9.6	0.02 *
Age group, n (%)				
≤49	38 (22.9)	98 (59.6)	136 (82.4)	0.11
≥50	4 (2.4)	25 (15.1)	29 (17.5)	
Civil status, n (%)				
Single	17 (10.2)	33 (20.5)	50 (30.3)	0.14
Married	25 (15.1)	75 (45.2)	100 (60.2)	
Divorced	0 (0)	6 (3.6)	6 (3.6)	
Living together	0 (0)	7 (4.2)	7 (4.2)	
Widower	0 (0)	2 (1.2)	2 (1.2)	
School level, n (%)				
Primary	0 (0)	6 (3.6)	6 (3.6)	<0.001 *
Secondary	4 (2.4)	56 (33.7)	60 (36.1)	
High school	12 (7.2)	42 (25.9)	54 (32.7)	
Degree	20 (12.0)	9 (5.4)	29 (17.5)	
Technical career	4 (2.4)	10 (6.0)	14 (8.4)	
Master’s degree	2 (1.2)	0 (0)	2 (1.2)	
Smoking, n (%)				
Smoker	6 (3.6)	19 (11.4)	25 (15.1)	0.04 *
Non-smoker	31 (18.7)	101 (61.4)	132 (80.0)	
Former smoker	5 (3.0)	3 (1.8)	8 (4.8)	
Family history of illness in close relatives (parents, brothers, and sisters), n (%)				
Obesity	15 (9.0)	35 (21.1)	50 (30.1)	0.36
Diabetes mellitus 2	16 (9.6)	59 (35.5)	75 (45.2)	0.28
Dyslipidemias	5 (3.0)	23 (13.9)	28 (16.9)	0.32
Arterial hypertension	11 (6.6)	57 (34.3)	68 (41.0)	0.02 *
Heart disease	1 (0.6)	20 (12.0)	21 (12.7)	0.02 *
Physical activity, n (%)				
Active (150–300 min/week)	21 (50)	25 (20.16)	46 (27.8)	<0.001 *
Sedentary (<150 min/week)	21 (50)	98 (79.03)	114 (72.12)	

Data are presented in the form of numerical values and percentages; the X^2^ test was used, and a *p*-value < 0.05 is considered statistically significant. Values with * superscript are significantly different.

**Table 2:** Anthropometric, clinical, and biochemical parameters evaluated in the study participants according to their BMI classification.

Variables	Normal Weight (n = 34)	Overweight (n = 69)	Obesity (n = 58)	Total (n = 161)	*p*
Anthropometric					
BMI (kg/m^2^)	23.0 ± 1.6 ^a^	27.4 ± 1.4 ^b^	32.0 ± 1.2 ^c^	28.0 ± 3.6	0.001 *
Waist circumference (cm)	76.5 ± 6.2 ^a^	85.5 ± 7.4 ^b^	95.5 ± 10.4 ^c^	87.0 ± 10.9	0.001 *
Biochemical					
Glucose (mg/dL)	79.1 ± 7.9	76.0 ± 7.0 ^c^	85.1 ± 24.9 ^c^	79.5 ± 16.5	0.005 *
Cholesterol total (mg/dL)	172.5 ± 38.4	188.0 ± 41.8	182.2 ± 34.0	182.8 ± 38.6	0.161
HDL-c (mg/dL)	54.9 ± 8.7 ^ab^	47.2 ± 11.0 ^a^	45.9 ± 15.0 ^b^	48.2 ± 12.5	0.005 *
LDL-c (mg/dL)	99.4 ± 36.7	116.0 ± 37.8	111.7 ± 31.5	110.9 ± 35.7	0.08
Triglycerides (mg/dL)	93.8 ± 43.2 ^b^	119.6 ± 49.5 ^c^	161.5 ± 97.2 ^bc^	129.2 ± 74.0	0.001 *
LDL/HDL-c ratio	1.89 ± 0.80 ^ab^	2.60 ± 1.06 ^a^	2.60 ± 0.96 ^b^	2.44 ± 1.01	0.003 *
Clinical (mm /Hg)					
Systolic blood pressure	111.5 ± 9.9	115.4 ± 14.7	117.8 ± 13.7	115.4 ± 13.5	0.080
Diastolic blood pressure	66.1 ± 6.7 ^b^	67.7 ± 9.0 ^c^	71.7 ± 10.4 ^bc^	68.7 ± 9.3	0.009 *

Abbreviations: BMI, body mass index; HDL-c, high-density lipoproteins; LDL-c, low-density lipoproteins. Data expressed as mean ± standard deviation. *p*-value calculated with ANOVA statistical test and Bonferroni post hoc test. *p* < 0.05 was considered significant, * indicates significant differences. Different superscript letters indicate significant differences in post hoc test. (^a^) normal weight vs. overweight; (^b^) normal weight vs. obesity; (^c^) overweight vs. obesity.

**Table 3:** Energy, macronutrients, dietary fiber, and cholesterol according to identified dietary patterns.

Variable	Mexican Fast-Food	Variety-Food	Healthy-Economic	*p*
Energy (kcal/d)	3166 ± 1185 ^a^	2762 ± 852 ^b^	2624 ± 827 ^b^	0.01 *
Carbohydrates (%)	48.53 ± 4.28 ^b^	49.46 ± 4.35 ^a^	47.15 ± 5.84 ^b^	0.05 *
Sugar (%)	5.87 ± 4.07	5.75 ± 3.69	4.93 ± 4.14	0.41
Protein (%)	13.48 ± 2.80	13.55 ± 1.61	14.27 ± 2.76	0.17
Lipids (%)	38.00 ± 4.17	36.99 ± 3.75	38.57 ± 5.05	0.16
Saturated fatty acids (%)	31.26 ± 5.99 ^a^	27.97 ± 4.47 ^b^	27.21 ± 4.83 ^b^	0.001 *
Monounsaturated fatty acids (%)	26.26 ± 3.60	27.31 ± 3.85	26.35 ± 4.92	0.31
Polyunsaturated fatty acids (%)	18.42 ± 4.40 ^a^	19.93 ± 4.31 ^b^	22.65 ± 5.00 ^c^	0.001 *
Dietary cholesterol (mg)	366.54 ± 157.63 ^a^	285.01 ± 95.77 ^b^	383.52 ± 251.53 ^c^	0.01 *
Dietary fiber (g)	23.24 ± 12.98 ^a^	27.94 ± 15.77 ^b^	22.56 ± 6.00 ^a^	0.05 *

Values are determined by the total energy value of the diet. Data presented as mean ± standard deviation, *p*-values calculated with ANOVA test and Bonferroni post hoc test. *p* < 0.05 was considered significant, * indicates significant differences. Different superscript letters indicate significant differences in post hoc tests. (^a^) Mexican Fast-Food vs. Healthy-Economic; (^b^) Mexican Fast-Food vs. Variety-Food; (^c^) Variety-Food vs. Healthy-Economic.

**Table 4:** Odds Ratios (ORs) and 95% confidence intervals (95% CI) of the scale of cardiovascular disease risk factors, dietary patterns, physical activity, and smoking habits in 165 Mexican adults.

				(95% CI for OR)	
Cardiovascular Disease Risk Factors Scale	B (ES)	*p*	Odds Ratio	Lower	Higher
Physical activity	1.5 (0.37)	<0.001 *	4.85	2.32	10.15
Smoking habits	1.8 (0.48)	<0.001 *	6.21	2.40	16.09
Adherence to the Mexican Fast-Food DP	0.59 (0.25)	0.032 *	1.71	1.04	2.80
Adherence to the Variety-Food DP	0.16 (0.29)	0.570	1.18	0.65	2.12
Adherence to the Healthy-Economic DP	−0.34 (0.34)	0.310	0.70	0.36	1.39

* Logistic regression model and generalized linear model, 95% CI. Variable scale of cardiovascular disease risk factors under 1–2 risk conditions; moderate 2–3 risk conditions; ≥4 risk conditions. Risk conditions: BMI (normal weight, overweight, obesity), abdominal obesity (≥80 for women; ≥90 for men), LDL/HDL-c ratio (≥2 with risk/≥1.9 without risk), triglycerides (≤149 mg/dL = desirable, ≥150 mg/dL = elevated), smoking (Smoker, Non-smoker), physical activity (active, sedentary).

## Data Availability

The datasets generated during and/or analyzed during the current study are available from the corresponding author upon reasonable request.

## Supplementary Materials

The following supporting information can be downloaded at https://www.mdpi.com/article/10.3390/nu16060804/s1. Figure S1. Flow-chart of the participants included in this study; Table S1. Factor-loading matrix for four major dietary patterns identified by principal component analysis; Table S2. Adherence to dietary patterns and sociodemographic characteristics, BMI, abdominal obesity, and lipid concentrations in Mexican adults; Table S3. Food groups for multivariate analysis of principal components.
