# Supplementary material for: Association of Dietary Patterns with Cardiovascular Disease Risk Factors in Mexican Adults: Insights from a Cross-Sectional Descriptive Study
Source: Nutrients. 2024 Mar 12;16(6):804. doi: 10.3390/nu16060804 (PMC10975449; doi:10.3390/nu16060804)
Supplement: Supplementary file 1 [file nutrients-16-00804-s001.zip › nutrients-2880755-supplementary.pdf]

Table S1. Factor-loading matrix for four major dietary patterns identified by principal component analysis.

Factor-loading matrix			
**Group of food**	**Mexican fast food**	**Variety-Food**	**Healthy-Economic**
Vegetables		**0.30**	**0.58**
Fruit		**0.67**	
Mature and fresh cheeses		**0.36**	
Oilseeds		**0.65**	
Monounsaturated oil and avocado		**0.64**	
Saturated and vegetable fat		**0.37**	
Polyunsaturated oils		**-0.33**	**0.34**
Dairy with sugar	**0.37**	**0.38**	
Mexican dishes tortillas and corn	**0.40**	**0.30**	
Sugary drinks	**0.38**		
Bread products wheat and rice	**0.55**		
Industrialized confectionery and sweet bread	**0.56**		
Industrialized foods and pizza	**0.68**		
Sweet foods	**0.53**		
Dairy	**0.55**		
Red and processed meats	**0.43**		
White meat and egg			**0.51**
Legumes			**0.60**
Fish and shellfish			**0.57**
Low-calorie drinks and water			**0.59**
**Total variance (31.62)**	**11.5 %**	**10.39%**	**9.72%**

Table S2. Adherence to dietary patterns and sociodemographic characteristics, BMI, abdominal obesity, lipid concentrations in Mexican Adults.

Variables	Mexican Fast-Food 58 (35.2%)	Variety-Food 54 (32.7%)	Healthy-Economic 53 (32.1%)	*p*
Gender				
Male n (%)	18 (42.8)	13(30.9)	11 (26.2)	0.44
Female n (%)	40 (32.2)	41(33.0)	42 (33.8)	
Smoking				
Non-smoker n (%)	43 (32.5)	47 (35.6)	43 (32.5)	
Smoker n (%)	11 (44)	5 (20)	8 (32)	0.52
Former smoker n (%)	4 (50)	2 (25)	2 (25)	
BMI	27.52 ± 3.63	28.00 ± 3.79	28.81 ± 3.42	0.17
Prevalence of overweight /obesity				
Normal n (%)	14 (38.8)	13 (36.2)	9 (25)	0.58
Overweight /obesity n (%)	44 (34.1)	41(31.8)	44 (34.1)	
Waist circumference (cm)	85.6 ± 9.2	87.3 ± 13.0	88.4 ± 10.3	0.41
Prevalence abdominal obesity				
Normal n (%)	41 (38.3)	37 (34.5)	39 (36.4)	0.13
Abdominal obesity n (%)	15 (27.7)	16 (29.2)	23 (42.6)	
Glucose (mg/dL)	77 ± 6	83 ± 25	79 ± 12	0.09
Cholesterol (mg/dL)	183 ± 40	180 ± 37	186 ± 39	0.71
HDL-c (mg/dL)	47 ± 10	48 ± 11	50 ± 16	0.37
LDL-c (mg/dL)	107.6 ± 34.8	111.0 ± 38.0	114.6 ± 34.8	0.60
Triacylglycerols (mg/dL)	135 ± 90	131 ± 64	121 ± 65	0.63
Risk LDL/HDL-c ratio				
No-risk n (%)	11 (28.9)	16 (47)	11 (28.9)	0.40
Risk n (%)	44 (36)	37(30.3)	41(33.6)	

Categorical variables are presented as numbers and percentages. For qualitative variables, the X2 test was used and for quantitative variables, one-way ANOVA was used. BMI (normal weight 18.0-24.9; overweight/obesity 25.0-34.9), Abdominal Obesity (⩾ 80 for women; ⩾ 90 for men), Abbreviations: BMI, body mass index; HDL-C, high-density lipoprotein; LDL-c, low-density lipoprotein.

Table S3. Food groups for multivariate analysis of principal components.		
	Groups	Food included in each group
1	Vegetables, seasonings, and spices	Swiss chard, spinach, purslane, cabbage, cauliflower, broccoli, lettuce, endive, red and green tomato or tomato sauce, carrot, squash and squash flower, green beans, eggplant, zucchini or cucumbers, peppers, asparagus, artichoke, leek, celery, cardon, onion, mushrooms, hot peppers, jalapeño, serrano, habanero, cooked nopales, chayote, jicama, poblano pepper. Garlic, parsley, thyme, bay leaf, oregano, coriander, salt.
2	Fruits	Orange, grapefruit, tangerine, lime, lemon, banana, apple, strawberries, cherries, plums, peach, apricot, nectarine, watermelon, melon, kiwi, grapes, fruits in syrup, dried dates, prunes, mango, guava, prickly pear, tamarind, papaya, and pineapple.
3	Milk	Nonfat milk, nonfat yogurt, semi-skimmed milk, whole milk, and whole yogurt.
4	Milk with sugar	Industrialized milkshakes, petit suisse type cheese, custard, flan, Jericaya, ice cream, fermented milk drink, condensed milk, atole.
5	Mature cheeses and fresh	Mature cured cheeses, Manchego, Gouda, Oaxaca<br>Fresh cheeses, jocoque cottage cheese, cottage cheese.
6	Legumes	Lentils, kidney beans, chickpeas, peas, and broad beans.
7	Oilseeds	Almonds, peanuts, hazelnuts, nuts, and pecans nuts.
8	Potatoes and snacks	Commercial french fries, churros, popcorn, french fries, fried peanuts, seeds, and beans.
9	White meat and eggs	Fat-free chicken, chicken with skin, egg
10	Red meats and processed meats	Beef, pork, liver, hamburger meat, meatballs, other organ meats, carnitas, ham. Processed meats (salami, chorizo, sausage).
11	Fish and other seafood	White fish, blue fish, salted fish, oysters, clams, mussels, squid, octopus, crustaceans, shrimp, canned tuna in water and oil, and canned tuna in tomato or pickle.
12	Monounsaturated oils and avocado	Olive oil, extra-virgin olive oil, avocado.
13	Saturated fats and vegetable shortening	Cream or cow cream, bacon, butter, lard, chicharrón, cream cheese, margarine, vegetable shortening.
14	Polyunsaturated oils	Corn oil, sunflower oil, soybean oil, safflower oil, canola oil.
15	Bread and products based on wheat and rice	Box bread, breakfast cereals, whole grains, bolillo cookies, pasta, noodles, macaroni, hamburger bread, flour tortilla, Maria cookie and rice.
16	Industrialized pastry and sweet bread	Industrialized donuts, cake, chocolate cookies, industrialized bread (cinnamon rolls, choco rolls), and

		sugary churros. Sweet bread (shells, palms, bows, semas, and muffins).
17	Industrialized foods and pizza	Packet soups and creams, mustard, mayonnaise, bottled. hot sauces, fried tomato sauce, ketchup sauce and pizza
18	High sugar foods	Cocoa powder for milkshakes, ate, dulce de leche, jam, cajeta, sweets, gummies, jellies, chocolates, sweets, sugar, piloncillo, honey.
19	Low-calorie/free drinks and water	Light carbonated drinks, decaffeinated coffee, espresso coffee, tea, soluble coffee, decaffeinated, and water
20	Sugary drinks	Carbonated beverages with sugar, plain orange juice, grapefruit juice, plain tangerine, bottled fruit juice, fruit water or flavor.
21	Alcoholic beverages	Beer, whiskey, vodka, gin, cognac, tequila.
22	Mexican dishes and tortillas and corn	Toast, tacos, pozole, tamales, mole, tortillas, and corn.

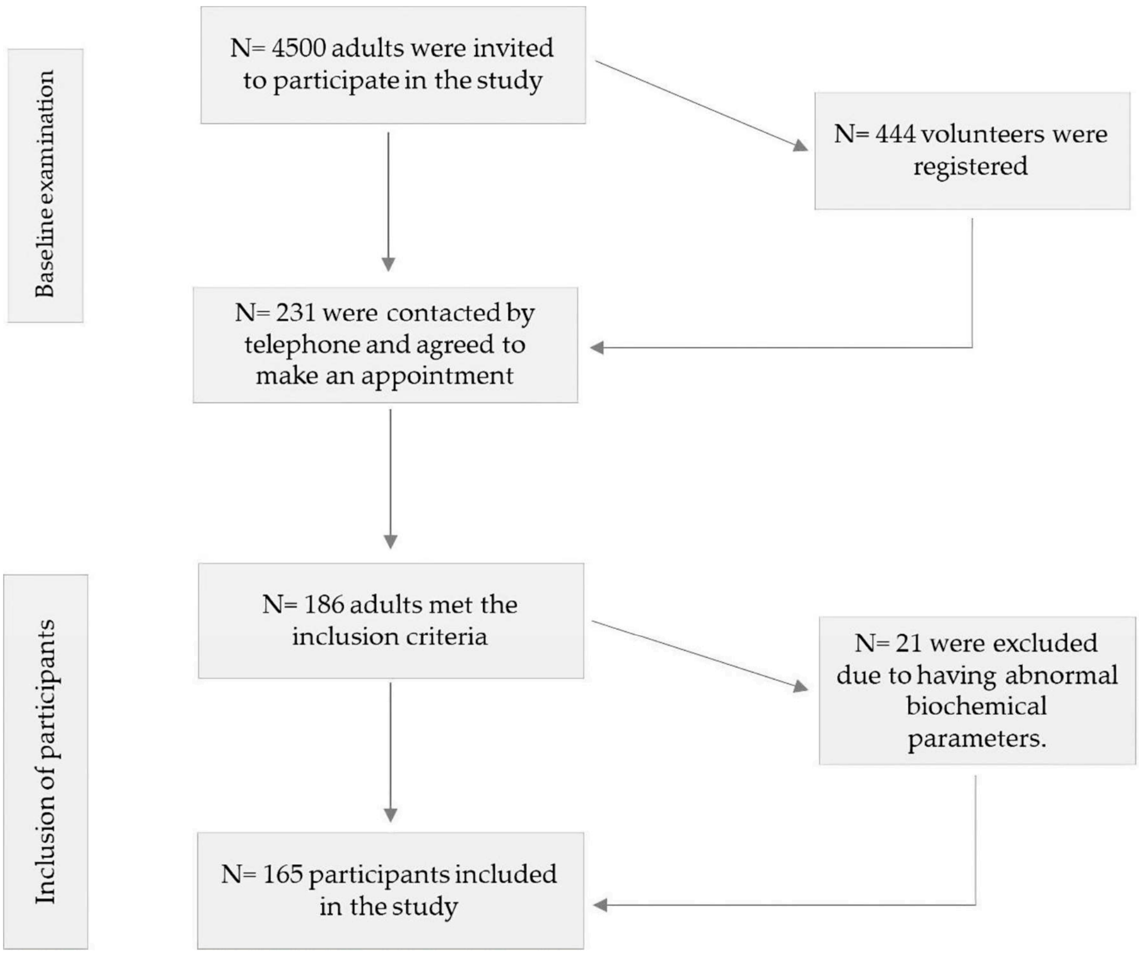

Figure S1. Flow-chart of the participants include in the study.
